# Unilateral retinoblastoma with contralateral isolated choroidal Melanocytosis: case report of an unexpected presentation

**DOI:** 10.1186/s12886-018-0916-x

**Published:** 2018-09-17

**Authors:** Cassanda C. Brooks, James J. Augsburger, Zélia M. Correa

**Affiliations:** 1 Department of Ophthalmology, University of Cincinnati College of Medicine, Cincinnati, OH USA; 2 Department of Ophthalmology, Duke University, Durham, NC USA; 3 Wilmer Eye Institute, Johns Hopkins University, 600 North Wolfe Street, Maumenee 711, Baltimore, MD 21287 USA

**Keywords:** Retinoblastoma, Unilateral, Ocular Melanocytosis, Melanoma, Uveal

## Abstract

**Background:**

Congenital ocular melanocytosis has been shown to be extremely uncommon in studies of numerous infants and children with retinoblastoma and disorders such as retinopathy of prematurity.

**Case presentation:**

A 33-month-old Caucasian boy presented with a solid white predominantly endophytic retinoblastoma filling most of the nasal aspect of the fundus and extensive vitreous seeding. Fundus exam of the contralateral eye showed a broad-based flat melanotic area of the choroid extending from the subfoveal region to the ora serrata temporally. The child was treated by enucleation of the retinoblastoma-containing eye (homozygous non-germline RB1 mutation) and is being monitored annually. The patient has been followed for 4 years.

**Conclusions:**

This rare presentation of advanced unilateral retinoblastoma and contralateral isolated choroidal melanocytosis in a young child emphasizes the importance of detailed fundus mapping of the non-affected eye and has potential implications due to the increased incidence of uveal melanoma later in life.

## Background

Retinoblastoma is the most common pediatric neoplasm of the eye and it accounts for 2.5% to 4% of all pediatric cancers [1]. The incidence of retinoblastoma is approximately 11.8 per million children aged 0–4 years US [2]. This cancer has 2 distinct clinical forms: bilateral or multifocal, heritable form (25% of all cases), and unilateral or unifocal form (75% of all cases), 90% of which are nonhereditary. However, about 10% of germline cases can be unilateral and unifocal. Although patients with germline disease have an increased risk to develop skin melanoma, there is no established relationship between retinoblastoma and uveal melanoma [1].

Due to the rarity of ocular melanocytosis and the documented increased incidence of uveal melanoma in eyes with complete ocular melanocytosis [3], we thought this unprecedented association deserves to be reported.

## Case presentation

**Fig. 1:**
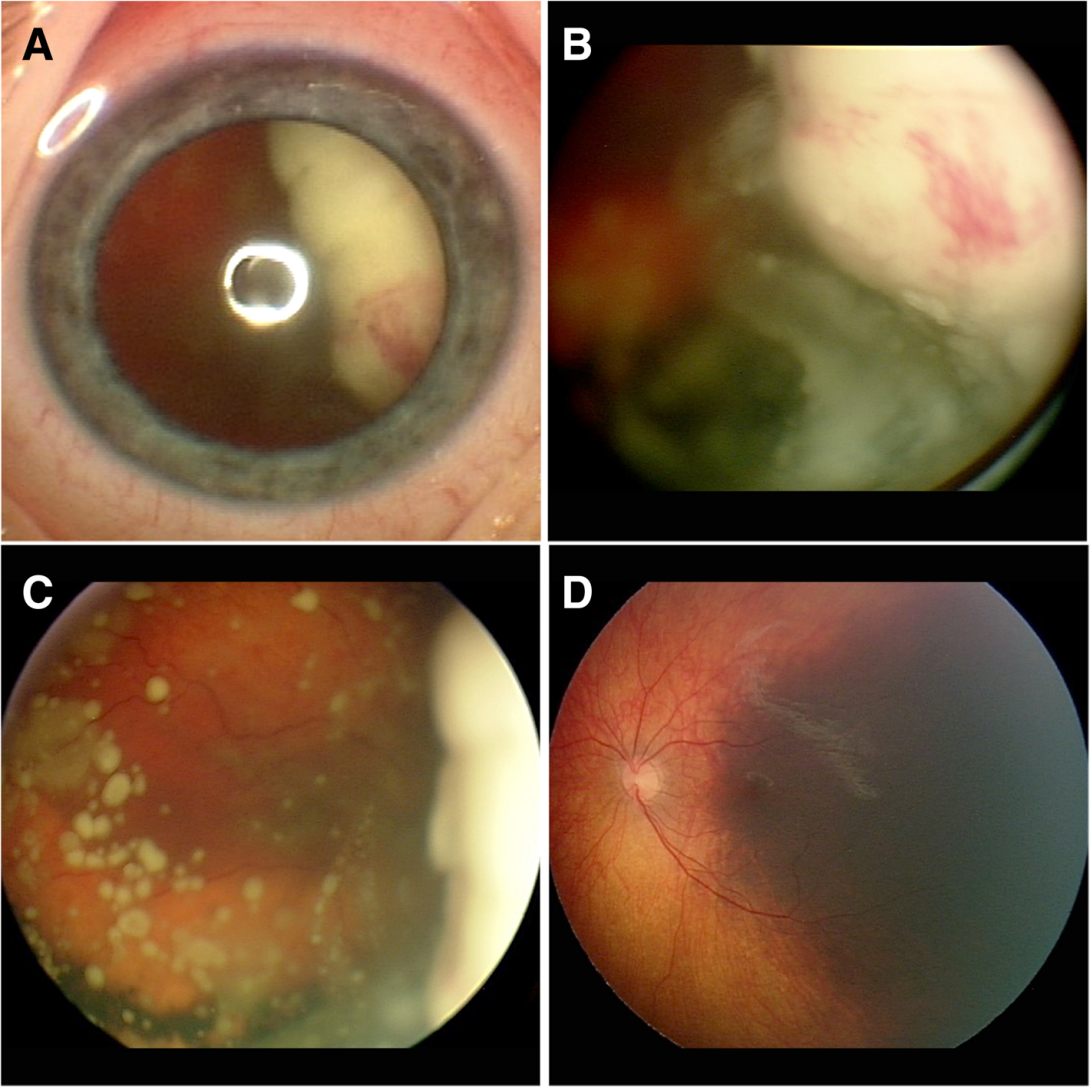
External (**a**) and fundus images (**b,c**) OD demonstrating solid white, predominantly endophytic retinal tumor with preretinal neovascularization on its surface (**b**) and extensive vitreous seeds (**b**, **c**). Fundus image OS (**d**) demonstrating broad-based flat melanotic area of choroid extending from the subfoveal region temporally

A 33-month-old Caucasian boy presented with leukocoria right eye (OD). Fundus examination OD revealed a solid white predominantly endophytic retinal tumor filling most of the nasal aspect of the fundus (Fig. 1a & b) and extensive vitreous seeding (Fig. 1c). The tumor extended to the posterior surface of the lens and exhibited preretinal neovascularization on its surface. B-scan ocular ultrasonography OD revealed dense intralesional particles consistent with calcific foci. Genetic testing demonstrated a homozygous non-germline RB1 nonsense mutation. Fundus examination of left eye (OS) revealed a broad-based flat melanotic area of the choroid extending from the subfoveal region to the ora serrata temporally (Fig. 1d). B-scan ocular ultrasonography OS showed no appreciable choroidal thickening corresponding to the melanotic patch. Anterior segment evaluation OS showed no iris or scleral melanocytosis. Our diagnoses were unilateral nonfamilial retinoblastoma OD and isolated choroidal melanocytosis^1^ OS. The child was treated by primary enucleation of the retinoblastoma-containing OD. Histopathologic evaluation confirmed the clinical diagnosis of retinoblastoma. The child has been followed for more than 4 years post-enucleation. Follow-up examinations of the fundus OS have shown no change in the patch of choroidal melanocytosis.

## Discussion and conclusions

Isolated choroidal melanocytosis (also known as sectoral choroidal melanocytosis) is a limited form of congenital ocular melanocytosis [3]. Examinations of numerous infants and children with retinoblastoma and disorders such as retinopathy of prematurity have shown such lesions to be extremely uncommon. Incidence of the broader category of ocular melanocytosis has been reported to be 0.038% in Caucasians in the US, however, no incidence reports are available for the isolated choroidal form [4]. Currently, no research indicates a correlation between retinoblastoma and isolated choroidal melanocytosis.

Complete ocular melanocytosis is recognized to be an important risk factor for development of uveal melanoma [5]. We suspect but cannot prove that isolated choroidal melanocytosis may predispose the affected eye to development of choroidal melanoma later in life, although at a lower frequency than the rate encountered with the complete form. Because of this risk, we are recommending that our patient be monitored at least once yearly, though he is currently examined twice yearly (once by her local ophthalmologist and once by our group).

This rare presentation of advanced unilateral retinoblastoma and contralateral isolated choroidal melanocytosis in a young child emphasizes the importance of detailed fundus mapping of the non-affected eye and has potential implications due to the increased incidence of uveal melanoma later in life.
